# Behavior Modeling for a Beacon-Based Indoor Location System

**DOI:** 10.3390/s21144839

**Published:** 2021-07-15

**Authors:** Aritz Bilbao-Jayo, Aitor Almeida, Ilaria Sergi, Teodoro Montanaro, Luca Fasano, Mikel Emaldi, Luigi Patrono

**Affiliations:** 1 DeustoTech Institute of Technology, University of Deusto, Av. Universidades 24, 48007 Bilbao, Spain; aitor.almeida@deusto.es (A.A.); m.emaldi@deusto.es (M.E.); 2 Department of Engineering for Innovation, University of Salento, Via Monteroni snc, 73100 Lecce, Italy; ilaria.sergi@unisalento.it (I.S.); teodoro.montanaro@unisalento.it (T.M.); luca.fasano@studenti.unisalento.it (L.F.); luigi.patrono@unisalento.it (L.P.)

**Keywords:** ambient assisted living, indoor positioning, internet of things, performance, smartphone, smartwatch, wearable device

## Abstract

In this work we performed a comparison between two different approaches to track a person in indoor environments using a locating system based on BLE technology with a smartphone and a smartwatch as monitoring devices. To do so, we provide the system architecture we designed and describe how the different elements of the proposed system interact with each other. Moreover, we have evaluated the system’s performance by computing the mean percentage error in the detection of the indoor position. Finally, we present a novel location prediction system based on neural embeddings, and a soft-attention mechanism, which is able to predict user’s next location with 67% accuracy.

## 1. Introduction

The advances in hardware and software technologies have led to the adoption of smart-environments in many contexts of our daily lives. Smart homes and smart buildings are already equipped with a multitude of embedded devices, along with connected sensors and actuators [1]. Several real cases already exemplify smart cities, which use the opportunities provided by innovative technologies to improve the lives of their inhabitants [2]. In such settings, smart environments are expected to play a crucial role for coping with the needs of sustainability, energy distribution, mobility, health and public safety/security [3]. A particular focus is the realization of ambient assisted living (AAL) solutions to enable elderly people to live independently for as long as possible, without intrusiveness from others. These solutions benefit from Internet of Things (IoT)-enabling technologies to improve elderly life thanks to the introduction of intelligent, connected devices [4].

Several AAL applications have been developed that have user positioning as their core capability. Elderly care [5], guidance systems [6], energy consumption [7] and security [8] are only some of the possible applications of indoor positioning information. Based on indoor positioning, it is possible to identify where a user is located and to predict his/her future locations based on the recent location history. In this paper, the indoor positioning issue is addressed by considering the performance obtained while using two different kinds of device to estimate the indoor position: a smartphone and a smartwatch. With both devices, the Bluetooth Low Energy (BLE) technology was exploited to obtain indoor positioning information. A generic home has been equipped with BLE beacon infrastructure, and several tests have been carried out with different configurations in terms of the number and models of beacons in each room. For each test campaign, the performance in terms of mean percentage error in the detection of the indoor position was calculated using a smartphone and a smartwatch, and the results have been discussed. For the location prediction, we present an algorithm based on using neural embeddings to represent the locations of a house and an attention-based mechanism that instead of being applied to the hidden states of the neural network architecture is used to modify those embeddings.

The rest of the paper is structured as follows. Section 2 contains an analysis of the state of the art. In Section 3 we describe the overall architecture of the system and in Section 4 the location prediction algorithm. Section 5 contains an explanation of the testing environment and we discuss the results of the experiments in Section 6. Finally, in Section 7 we draw the conclusions and propose future areas of research.

## 2. Related Work

### 2.1. Indoor Location

Indoor positioning systems (IPS) are an essential part of any intelligent environment or pervasive computing system. Indoor positioning has been used to model users’ behavior in order to detect early risks related to frailty in elders [9], guide museum visits [10] and coordinate emergency responses [11]. There are different approaches and technologies that have been proposed over the years to tackle indoor positioning. Vision-based approaches use either visible light systems [12] or infrared signals, such as the Active Badge Location System, wherein a wearable tag emits an infrared code that is captured by an interconnected network of sensors [13]. Other vision-based systems use computer vision to detect specifically generated bidimensional codes in order to locate users and devices in an intelligent environment, such as the TRIP location system [14]. In the context of ambient assisted living, in [15] a video-based monitoring system for elderly care was proposed. The main objectives of this system are to preserve elderly independence and increase the efficiency of the homecare practices. The main disadvantage of the vision-based technology lies in the cost, which is still too high, especially for systems with very high precision. Alternatives to these systems are the radio frequency-based systems, such as those using Wi-Fi [16], RFID or Bluetooth.

Radio frequency identification (RFID) is one of the most popular wireless technologies for tracing and positioning [17,18]. The main advantage of this technology is the capability to work in the absence of line of sight (LoS). An example of this is the work done in [19]. The authors used Bayesian probability and k-nearest neighbors in combination with RFID tags. Other authors applied a deep belief network as fingerprinting-based RFID indoor localization algorithm [20]. Additionally, a combination of hyperbolic positioning and genetic algorithms has been used in order to compute the phase offset caused by the interference between tags [21]. NFC systems, such as [22], can be considered a sub-category of RFID systems. In most cases, such systems have the drawback of requiring a smartphone to approach deployed beacon. This type of active participation from the users is not desirable in most scenarios.

Bluetooth technology is an alternative for indoor positioning [23]. It can guarantee a low cost since it is integrated in most of our devices that are used daily, such as tablets and smartphones. Moreover, the spread of the emerging BLE technology makes BT also energy efficient, which is a key requirement in many indoor applications. This efficiency allows for higher measuring rates when determining a user’s location and for longer battery life. For these reasons, BLE is considered as one of the most suitable positioning technologies for indoor positioning currently. The recent rise of iBeacons by Apple has contributed to the rapid spread of this technology, which is used to provide information and location services [24] in a completely innovative way. The accuracy of BLE for indoor locating has been extensively studied by several authors [25]. Subedi et al. [26] proposed the use of weighted centroid localization alongside the received signal strength indications from the neighboring BLE beacons. However, in order to achieve similar accuracy rates to Wi-Fi based approaches, BLE beacon-based approaches require more beacons than Wi-Fi APs [27].

Ultra-Wide Band (UWB) is another alternative for accurate indoor positioning. García et al. [28] presenteded a novel system for indoor positioning using UWB in highly complex environments where there are non-line-of-sight (NLOS) conditions. To do so, the authors used an extender Kalman filter for a NLOS detection algorithm. UWB has been widely applied in the tracking of sports activities in indoor environments [29,30]. A more extensive analysis of the state-of-the-art can be found in the following reviews: [31,32].

### 2.2. Behavior Prediction and Modeling

User behavior prediction and modeling is an area of research applied to several domains. As discussed in [33], behavior prediction is a core problem to be solved in the creation of more energy efficient and sustainable spaces. In [34], the authors applied behavior prediction to the online behavior in order to identify malicious users. In [35] it was used for marketing purposes. Behavior prediction is a commonly used technique in both real and virtual intelligent spaces. In [4], behavior was used to identify the risks related to mild cognitive impairments and frailty in the elderly in IoT-augmented spaces. The authors of [36] used behavior prediction to create more intelligent automated homes. In [37], the authors used similar behavior predicting approaches, but in this case to predict the behavior in virtual spaces.

Different techniques have been used to tackle this problem. Almeida et al. [38,39] have studied the usage of both convolutional neural networks (CNNs) and long short-term memory (LSTMs) architectures to predict users’ behavior while representing actions with neural embeddings. A LSTM approach was also used in [40] to learn and predict design commands based upon building information modeling (BIM) event log data stored in Autodesk Revit journal files. In [41] the authors followed a neuro-fuzzy approach. A Gaussian radial basis function neural network (GRBF-NN) was trained based on the example set generated by a fuzzy rule-based system (FRBS) and the 360-degree feedback of the user. Kim et al. [42] studied using RNN architectures in order to predict multi-domain behavior. In [43], the authors used both attention and memory mechanisms in their neural network architectures to improve the prediction results.

## 3. System Architecture

**Figure 1:**
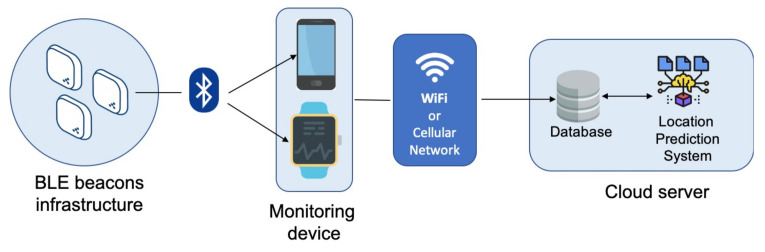
The system’s overall architecture.

In Figure 1, the system’s overall architecture is depicted. It mainly consists of the following components:

- BLE beacon infrastructure.
- A monitoring device to capture positioning data.
- A cloud server to store and process captured data.

Beacons are small radio transmitters that send Bluetooth signals. They are available in different sizes and shapes, making them suitable for a wide range of applications and allowing them to be easily integrated into any environment unobtrusively. A beacon is cost-effective and can be installed easily, and its position can be determined with to within a few meters. The BLE standard is also very energy efficient. Beacons can be used in server-based (asset tracking) and client-based (indoor navigation) applications. The last option was used in our study. Specifically, in the proposed solution, the indoor environment is equipped with a BLE beacon infrastructure. In particular, a BLE beacon is placed in each room, but in large rooms or long corridors, more beacons can be placed.

On the server side, every association between a beacon (i.e., the MAC address of the beacon) and its location (i.e., the room in which it is located) is stored in the database. When the application starts, this beacon/room map is transmitted from the server to the local database on the monitoring device. In this way, the application performs preliminary filtering during the scanning phase and only considers signals from beacons that are part of the implemented infrastructure for subsequent operations. The monitoring device consists of a smartphone or smartwatch running a specially designed and implemented application. In particular, the mobile application performs repeated Bluetooth scanning in configurable time intervals. With our settings, Bluetooth scanning lasts 10 seconds, and the next scan starts 15 s after the end of the previous one. During the scanning phase (i.e., within a 10-s interval), each beacon will be detected multiple times, triggering an event. Specifically, the average value of the detected RSSI and the average value of the transmission power (TxPower) (the power at which the beacon broadcasts its signal) are calculated. At the conclusion of the scanning process, a list of beacons identified by MAC address is obtained, along with relative average RSSI and TxPower values. Using these values, the calculateRating function in Listing 1 is applied for each beacon.

**Listing 1.** Function used to calculate the RSSI accuracy.
1


/*


2


* Calculates the accuracy of RSSI value considering txPower


3


* https://developer.radiusnetworks.com/2014/12/04/fundamentals-


4


* beacon-ranging.html


5


*/


6

protected static double calculateRating(int txPower, double rssi) {

7

  if (rssi == 0) {

8

    return -1.0; // if we cannot determine accuracy, return -1.

9

  }

10


11

  double ratio = rssi*1.0/txPower;

12

  if (ratio < 1.0) {

13

    return Math.pow(ratio,10);

14

  }

15


  else


16

    return (0.89976)*Math.pow(ratio,7.7095) + 0.111;

17

}


This allows an “accuracy” value, called a rating, to be assigned to each beacon, which is used to correct the detected average RSSI value.

The formula used in the previous code to calculate the rating was [44]:

(1) $$rating=0.89976\cdot {\left(\frac{rssi}{txPower}\right)}^{7.7095}+0.111$$

The three constants in the formula (0.89976, 7.7095 and 0.111) are based on a best fit curve based on a number of measured signal strengths at various known distances from a Nexus 4. However, because the accuracy of this measurement is affected by errors, our algorithm uses the formula generically as a “rating” rather than as a true distance.

Additionally, each beacon is linked to an indoor location (room) via the function in Listing 2.

**Listing 2.** Function used to link each beacon to the corresponding room.
1


/*


2


* IndoorLocation


3


* @param beacon_address       the BLE beacon mac address


4


* @param location_type        the location (room) string label


5


* @param location_id         the location (room) specific beacon identifier


6


* @param location_calibrated_rssi  the integer value of RSSI measured at 1 meter


7


*/


8

indoorLocations.add(new IndoorLocation("E0:2E:61:8A:19:E7", "Livingroom", "53", -70));

9

indoorLocations.add(new IndoorLocation("D3:5E:63:38:3B:45", "Bedroom", "56", -65));

10

indoorLocations.add(new IndoorLocation(DC:64:4C:44:61:8C", "Livingroom", "32", -50));

11

indoorLocations.add(new IndoorLocation("D4:64:95:34:4F:46", "Bathroom", "LVR", -70));

12

indoorLocations.add(new IndoorLocation("FD:19:B1:2A:45:6B", "Bathroom", "46", -80));

13

indoorLocations.add(new IndoorLocation("F0:A9:05:0A:6F:DB", "Bathroom", "54", -60))

14

indoorLocations.add(new IndoorLocation("C8:7E:EC:5F:E4:00", "Bedroom", "47", -60));

15

indoorLocations.add(new IndoorLocation("D4:3E:77:B1:F8:9D", "Kitchen", "45", -50));

16

indoorLocations.add(new IndoorLocation("E9:68:B7:2C:F9:68", "Kitchen", "23-black", 0));


As a result, each beacon is identified by its MAC address, associated with the corresponding room (e.g., living room, bathroom, bedroom or kitchen), and labeled with a location ID label (53, 56, 32, LVR, etc.) In addition, each beacon has a calibration RSSI value that corresponds to the average RSSI value measured at a 1 meter distance.

Finally, the distance from each beacon is calculated using this calibration value and the “log-distance path loss” [45] as reported in Listing 3.

**Listing 3.** Distance calculation.
1


/**


2


 * Calculates distances using the log-distance path loss model


3


 * 


4


 * @param rssi      the currently measured RSSI


5


 * @param calibratedRssi  the RSSI measured at 1m distance


6


 * @param pathLossParameter the path-loss adjustment parameter


7


 */


8

public static double calculateDistance(double rssi, float calibratedRssi) {

9

float pathLossParameter = 3f;

10

return Math.pow(10, (calibratedRssi - rssi) / (10 * pathLossParameter));

11

}


Then, the beacon list is sorted according to the rating value. Since the calculated rating is proportional to the distance, as specified in the formula (1), the first beacon in the list is the beacon with the lowest rating and closest to the smartphone.

The information about the nearest beacon is sent to the cloud server by the application. All detected locations are saved on the server and provided to the location prediction system to be processed, as described in Section 4.

## 4. Location Prediction System

**Figure 2:**
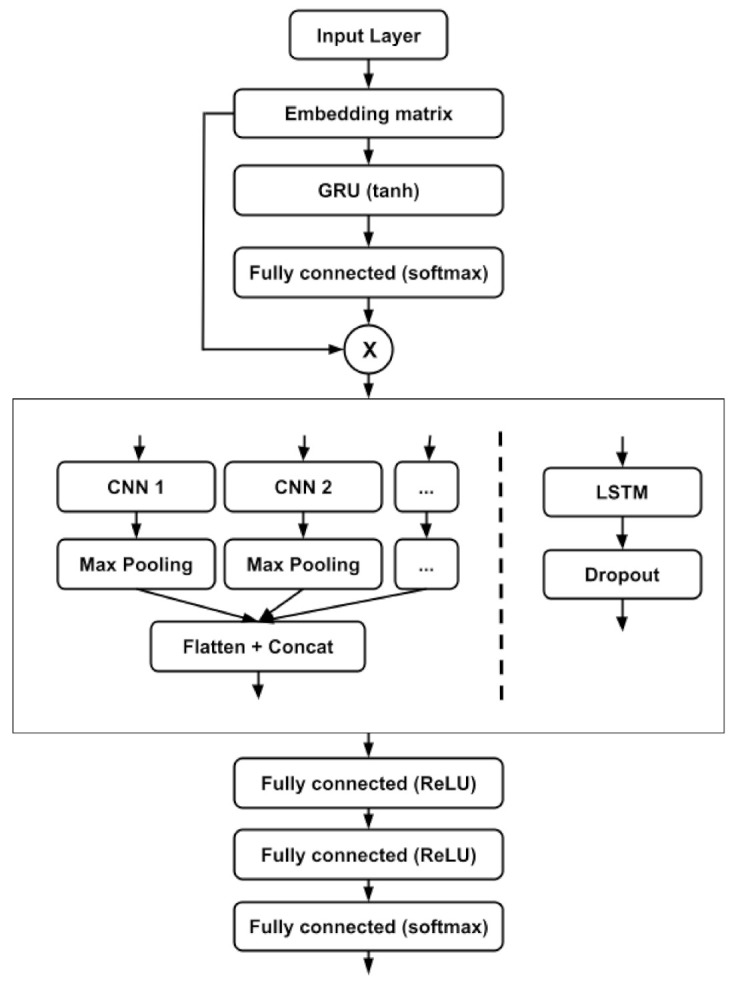
The architecture of the location prediction algorithm. Both approaches are shown in the same image, as they share the input, attention and output modules.

The location prediction system is based in our previous work [38,39] focused on predicting users’ behavior. The algorithm we present in this paper models the user’s movements through indoor locations; it uses the semantic location to model them. One of the characteristics of our algorithm is that it works in the semantic-location space instead of the sensor space, which allows us to abstract from the underlying indoor location technologies. The location prediction is divided into four modules that process the data sequentially (see Figure 2):

1. **Input module:** It takes the semantic locations as inputs and transforms them into embeddings to be processed. It has both an input and an embedding layer.
2. **Attention mechanism:** It evaluates the location embedding sequence to identify those that are more relevant for the prediction process. To do so, it uses a GRU layer, followed by a dense layer with a tanh activation and finally a dense layer with a softmax activation.
3. **Sequence feature extractor:** It receives the location embeddings processed by the attention mechanism and uses a 1D CNN or a LSTM to identify the most relevelant location n-grams of sequences of locations for the prediction. In case of the CNNs, multiple 1D convolution operations are done in parallel to extract n-grams of different lengths in order to obtain a rich representation of the relevant features.
4. **Location prediction module:** It receives the features extracted by the sequence feature extractor (multi-scale CNNs or LSTMs) and uses those features to predict the next location. This module is composed of two dense layers with ReLU activations and an output dense layer with a softmax activation.

### 4.1. Input Module

The input module is in charge of receiving the location IDs and using the embedding matrix to get the vectors that represent them. As we demonstrated in [38], using better representations, such as embeddings, instead of IDs, provides better predictions. The proposed system uses Word2Vec to obtain the embedding vectors [46], a model widely used in the NLP community.

Given a sequence of locations $S_{loc}=\left[l_{1},l_{2},\cdots ,l_{n}\right]$ where *n* is the length of the sequence and $a_{i}\in R^{n}$ indicates the location vector of the *i*th location in the sequence, and $Context\left(l_{i}\right)=\left[l_{i-n},\cdots ,l_{i-1},l_{i+1},\cdots ,l_{i+n}\right]$ represents the context of $l_{i}$, the window size being 2n. $p(l_{i}|Context\left(l_{i}\right))$ is the probability of $l_{i}$ being in that position of the location instance sequence. To calculate the embeddings, we try to optimize the log maximum likelihood estimation:

(2) $$L_{l}\left(MLE\right)=\sum_{\begin{array}{c} l_{i}\in l_{act} \end{array}}logp\left(l_{i}|Context\left(l_{i}\right)\right)$$

Our system uses Gensim to calculate the embedding vectors for each location in the dataset. The location embedding vectors have a size of 50. To translate the one-hot encoded location IDs to embeddings, we use an embedding matrix, instead of providing the embedding values directly. This allows us to train this matrix and adapt the calculated embeddings to the task at hand, thereby improving the results.

### 4.2. Attention Mechanism

Once we have the semantic embeddings for each location, they are processed by the attention mechanism to identify those locations in the sequence that are more relevant for the prediction process. To do so we use a soft attention mechanism. This is a similar approach to the ones used in NLP to identify the most relevant words in a phrase. However, we do something different in this approach: applying the attention mechanism to the embeddings instead of the hidden steps of the sequence encoder. As proven in [38], this approach has achieved better results when predicting locations.

Location sequences $S_{loc}$ are temporally ordered sets of locations $l_{t}$, given t∈[1,T]. The location sequence $l_{t},t\in \left[1T\right]$ goes through the input module, which uses the matrix $L_{e}$, calculated previously with Word2Vec, to obtain the location embedding vectors. Those embedding vectors are then processed by the gated recurrent unit layer, creating a representation of the sequence. This gated recurrent unit layer reads the location sequence from $l_{1}$ to $l_{T}$. The used gated recurrent unit layer has a total of 128 units.

(3) $$x_{t}=L_{e}l_{t}$$

(4) $$h_{t}=\vec{GRU}\left(x_{t}\right)$$

The attention module gets the gated recurrent unit’s layer states $h_{t}$ and creates a vector of weights $\alpha_{t}\in \left[0,1\right]$ with the relevance of each location $l_{t}$. This is done using a dense layer with a unit size of 128 to get the hidden representation $u_{t}$ of $h_{t}$:

(5) $$u_{t}=tanh\left(W_{l}h_{t}+b_{l}\right)$$

Then we use a softmax function to calculate the normalized relevance of the weights ($\alpha_{t}$) for the location instances:

(6) $$\alpha_{t}=\frac{exp(u_{t}^{⊤}u_{l})}{\sum_{t}exp\left(u_{t}^{⊤}u_{l}\right)}$$

The obtained vector is used to calculate the relevance of the location embeddings $x_{t}$ for the prediction, $L_{eadj}$:

(7) $$L_{eadj}=\alpha_{t}x_{t}$$

Those embeddings $L_{eadj}$ are the used to process the sequence.

### 4.3. Sequence Feature Extractor

After obtaining the attention modified location embeddings $L_{eadj}$ in Equation (7), we tested two different approaches to perform the feature extraction: CNNs and LSTMs.

On the one hand, the CNN architecture was used to extract the features of the sequence. This architecture was composed of multiple 1D CNNs that processed the sequence in parallel with different kernel sizes. This was done to identify differently sized n-grams in the location sequences. The location sequences had a set length: $L_{eadj}=\left\{le_{1},...,le_{lc}\right\}$. The size of the location embedding was represented by $d_{le}$, and the elements of the embedding were real numbers, $le_{i}\in {ℜ}^{d_{le}}$. After getting the attention modified location embeddings, each location sequence was represented like $L_{eadj}\in {ℜ}^{l_{le}\times d_{le}}$. The convolution operation was:

(8) $$O_{j}=f\left(W_{j}\circ \left[le_{1},...,le_{l_{le}-s+1}\right]+b\right)$$

The result of the operation was $O_{j}\in {ℜ}^{l_{le}-s+1}$, and $W_{j}\in {ℜ}^{l\times d}$, and *b* were the trained parameters. The activation function f() was a rectified linear unit, and $W\circ L_{eadj}$ represents the element-wise multiplication. Using $d_{o}$ filter maps, the output of the operation was $O=\left[O_{1},...,O_{d_{o}}\right]\in {ℜ}^{\left(l_{le}-s+1\right)\times d_{o}}$. Two hundred filters were used in this case for each convolution operation. After each convolution layer we used 1 max pooling layer. Finally, the results of all the parallel convolution layers were concatenated and flattened.

On the other hand, the LSTM with 512 units received the location embeddings and analysed the existing temporal relations among the different locations that formed each of the sequences. Then, dropout normalization was applied to the extracted features.

### 4.4. Location Prediction Module

The input for the location prediction module is the output of the previously described feature extractor module. To predict the most probable location, this module uses three dense layers. The first two ones ($f_{re}$) use rectified linear units as their activation:

(9) $$f_{re}=relu\left(WX+b\right)$$

To predict the location, the final dense layer uses a softmax activation. The output of this module is a vector with the probabilities of each possible location.

## 5. Test Environment

### 5.1. Physical Location

**Figure 3:**
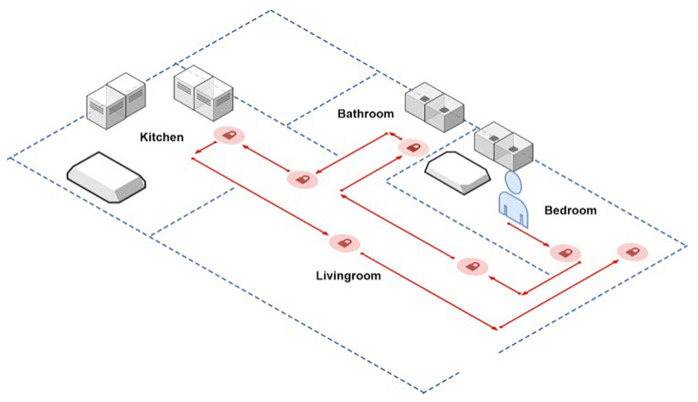
The test environment with the established path and checkpoints.

To assess the indoor positioning system’s performance, a realistic scenario was created in which the user wore a smartwatch or placed a smartphone in his or her pocket and moved around his or her home. The house measured 15 m × 7 m and had five rooms. A path was defined inside the house that led to four different rooms, each with one or more BLE beacon. The user started to follow the established path from the bedroom, as shown in Figure 3. One or more checkpoints were established for each room (indicated by the circle icon).

Three tests have been carried out, each being characterized by different BLE beacon infrastructure, and each test was repeated eight times. Both monitoring devices (smartphone and smartwatch) were used at the same time in each test to compare smartphone and smartwatch performance. The results of the indoor tracking method were read three times at each checkpoint, with a ten-second interval between each detection. The number of false positives was counted for each detection (a false positive occurred when the beacon detected by the mobile application was different from the beacon associated with the checkpoint).

The configuration used in each test was as follows:

1. **Test 1**
  Beacon model: the battery powered BlueBeacon Mini by BlueUp [47].
  Number of beacons: one BLE beacon in each room.
2. **Test 2**
  Beacons model: the AKMW-iB005N-SMA by AnkhMaway [48] with a USB power supply.
  Number of beacons: one BLE beacon in each room.
3. **Test 3**
  Beacons models: BlueBeacon Mini and AKMW-iB005N-SMA.
  Numbers of beacons: one AKMW-iB005N-SMA in the bedroom, one AKMW-iB005N-SMA in the bathroom, two BlueBeacon Mini in the Kitchen and two AKMW-iB005N-SMA in the living room.

### 5.2. Dataset

The dataset consists of location data of a single user gathered through a smartwatch over the course of a week. Each time a location change was detected by the smartwatch, the new location and timestamp were stored. In total, the dataset has 267 location changes and four different locations: bedroom, living room, bathroom and kitchen. For the training process, we split the dataset into a training set (80% of the dataset) and a validation set (20% of the dataset) of continuous days.

Since the model uses *n* previous locations (5 in this case) as input to predict the next location, the dataset was split into sequences of *n* locations, the next location being the one that the model has to predict. Therefore, the training set had 209 training samples, and there were 52 test samples.

## 6. Results

### 6.1. Indoor Location System

To evaluate the performance of the smartphone and the smartwatch in our indoor location system, the positions detected by the two monitoring devices during the performed tests were compared. For each test and for each iteration, the numbers of false positives at the checkpoints have been calculated. The percentage of error was calculated by the ratio between the number of total false positives and the number of total detections (21 total detections for each test repetition) by using the following equation:

(10) $$err_{perc}=100\cdot \frac{1}{n}\cdot \sum_{i=1}^{n}x_{i}$$

where *n* is the number of total detections and *x_i_* is the number of false positives at the i-th iteration (this value was 0 or 1). Moreover, for each test and for each iteration, the average absolute deviation (AAD) has been calculated by using the following equation:

(11) $$AAD=\frac{1}{k}\cdot \sum_{i=1}^{n}\left|y_{i}-\mu \right|$$

where *y_i_* is the number of false positives at the i-th iteration, μ is the average value of false positives and *k* is the total number of test repetitions (in our case, eight is the value to assign to the parameter *k*).

Table 1 presents the results of test 1. From this table, it is possible to notice that in this configuration the smartwatch ensured better performance (the mean percentage error was less compared to the mean percentage error obtained with the smartphone as a monitoring device).

Table 2 presents the results of test 2. In this case, a general improvement of the indoor localization method performance can be observed. The mean percentage error was reduced from 36.31% to 23.21% using the smartphone, and from 21.43% to 13.69% using the smartwatch. This is because the beacons used in this test had a wider transmission range (the maximum distance at which beacon’s signal can be received). In fact, though the transmission range depends of many factors (beacon installation position, operating environment and receiver performance, just to name a few), at the same TxPower of +4 dBm, the theoretical maximum distance (in Line of Sight free-space condition) offered by the AKMW-iB005N-SMA is 130 m, which is 100 m greater than for the BlueBeacon Mini. Additionally, in this configuration, the performance registered using the smartwatch as the monitoring device was better.

Finally, Table 3 presents the results of the test 3. In this test the mean percentage error using the smartphone was higher compared to the mean percentage error obtained in test 2 for the same monitoring device. This was due to the high reception capacity of the smartphone antenna, which was too sensitive if several beacons were placed close in the same environment. The mean percentage error using the smartwatch was reduced to 7.74%. It is possible to draw some conclusions based on the results of all three tests. Several factors, such as the positions of the beacons within the room (for example, height from the ground) or the distances between the beacons, can influence indoor tracking results. For example, beacons close to each other can cause interference. However, in general, the smartwatch guaranteed better performance in indoor localization than the smartphone with the same configuration in BLE beacon infrastructure.

### 6.2. Location Prediction System

We have evaluated the proposed approach by comparing our results in terms of accuracy score with two different approaches that have been used in the location prediction literature as baselines: nearest locations (NL) where the nearest neighbor to the user’s current location is selected [49], and a hidden Markov model which characterizes the movement patterns [50]. As can be seen in Table 4, our approaches outperformed the proposed baselines by a wide margin.

Moreover, to give more insights into the performance of the proposed architectures, we have evaluated the proposed location prediction system using the top-k accuracy score. This score measures how many times the ground truth (or correct label) is among the top *k* predicted labels provided by the fully connected layer with a softmax activation function.

$l_{i}$ is the correct location, $T_{i}^{k}$ the ordered list of the top *k* predicted locations, *N* the number of tests samples and *b* the scoring function with two possible outputs 0,1. The top-k accuracy is formulated in Equation (12):

(12) $$acc\_at\_k=\frac{1}{N}\sum_{i=1}^{N}b\left[l_{i}\in T_{i}^{k}\right]$$

Therefore, the value of the scoring function is 1 when the label of the correct location exists in the ordered list of the top *k* predicted locations. On the contrary, if the label is not in the ordered list, the function will return 0. For this experimentation, we report the accuracy scores with k=1, k=2 and k=3 since we had a total of four possible outcomes.

Table 5 presents the results. The best results for accuracy at 1 and 3 were obtained in experiment L2, where the LSTM with the previously mentioned attention mechanism was used. However, the best metric for accuracy at 2 was obtained in M2, where multi-scale convolutional neural networks were used with the same attention mechanism. In both cases, the best results were achieved using the embedding level attention mechanism introduced in [38]. However, in this dataset the best results overall were achieved using LSTMs.

## 7. Conclusions

In this paper we present an indoor locating system based on BLE and its evaluation utilizing a smartphone and a smartwatch as monitoring devices. Over that system, we built a behavior prediction system based on locations and validated two different approaches. Our system provides a holistic approach to an indoor location system, providing both the necessary infrastructure and the intelligent framework over it.

The system’s performance in terms of mean percentage error was assessed and analyzed. A distinct BLE beacon infrastructure was considered for each test by altering the quantity and models of BLE beacons in each room of the considered indoor environment. The best results were achieved using the smartwatch instead of the smartphone.

Furthermore, a position prediction system based on neural embeddings to represent the locations of a house was introduced, along with an attention-based mechanism that modifies those embeddings rather than having them applied to the hidden states of the neural network design. The location prediction system’s accuracy has also been assessed, compared with other approaches and discussed. From these experiments, two main conclusions can be drawn: first, the proposed attention mechanism applied to the embeddings improves the architecture’s performance; and second, despite the limited size of training samples, the presented deep neural network architectures performed better than shallow machine learning algorithms such as hidden Markov models.

The system will be expanded in the future by including RFID components, such as wearable RFID devices and RFID tags, to capture data that will be processed by an activity recognition module. From location data and RFID data, this module will be able to deduce user activities. Additionally, as a future extension of the presented work, it would be interesting to use algorithms such as the Kalman filter to improve the results of our indoor location system. Regarding the location prediction system, using the transformers introduced by Vaswani et al. [51] could improve its performance.

## Figures and Tables

**Table 1:** Test 1. Percentage error and false positives.

Test Repetition	Smartphone False Positives	Smartphone Percentage Error	Smartphone Absolute Deviation	Smartwatch False Positives	Smartwatch Percentage Error	Smartwatch Absolute Deviation
**1**	6	28.57%	1.625	4	19.05%	0.5
**2**	6	28.57%	1.625	4	19.05%	0.5
**3**	8	38.10%	0.375	6	28.57%	1.5
**4**	7	33.33%	0.625	4	19.05%	0.5
**5**	9	42.86%	1.375	2	9.52%	2.5
**6**	10	47.62%	2.375	6	28.57%	1.5
**7**	7	33.33%	0.625	3	14.29%	1.5
**8**	8	38.10%	0.375	7	33.33%	2.5
**Average**	**7.625**	**36.31%**	**1.125**	**5**	**21.43%**	**1.375**

**Table 2:** Test 2. Percentage error and false positives.

Test Repetition	Smartphone False Positives	Smartphone Percentage Error	Smartphone Absolute Deviation	Smartwatch False Positives	Smartwatch Percentage Error	Smartwatch Absolute Deviation
**1**	9	42.86%	4.125	3	14.29%	0.125
**2**	6	28.57%	1.125	3	14.29%	0.125
**3**	4	19.05%	0.875	3	14.29%	1.125
**4**	4	19.05%	0.875	1	4.76%	1.875
**5**	3	14.29%	1.875	2	9.52%	0.875
**6**	4	19.05%	0.875	2	9.52%	0.875
**7**	9	42.86%	4.125	5	23.81%	2.125
**8**	0	0.00%	4.875	4	19.05%	1.125
**Average**	**4.875**	**23.21%**	**2.344**	**3**	**13.69%**	**0.906**

**Table 3:** Test 3. Percentage error and false positives.

Test Repetition	Smartphone False Positives	Smartphone Percentage Error	Smartphone Absolute Deviation	Smartwatch False Positives	Smartwatch Percentage Error	Smartwatch Absolute Deviation
**1**	6	28.57%	0.25	3	14.29%	1.375
**2**	9	42.86%	3.25	1	4.76%	0.625
**3**	5	23.81%	0.75	2	9.52%	0.375
**4**	3	14.29%	2.75	3	14.29%	1.375
**5**	6	28.57%	0.25	2	9.52%	0.375
**6**	6	28.57%	0.25	0	0.00%	1.625
**7**	5	23.81%	0.75	1	4.76%	0.625
**8**	6	28.57%	0.25	1	4.76%	0.625
**Average**	**5.75**	**27.38%**	**1.063**	**2**	**7.74%**	**0.875**

**Table 4:** Accuracy scores of the performed experiments. Nearest locations (NL), hidden Markov models (HMM), multi-scale CNNs (M1), multi-scale CNNs with attention (M2), LSTM (L1), LSTM with attention (L2).

ID	Accuracy
NL	0.5
HMM	0.5961
M1	0.6538
M2	0.6538
L1	0.6346
L2	**0.6731**

**Table 5:** Top-k accuracy score results of the best performing experiments. Multi-scale CNNs (M1), multi-scale CNNs with attention (M2), LSTM (L1), LSTM with attention (L2).

ID	acc_at_1	acc_at_2	acc_at_3
M1	0.6538	0.9038	0.9423
M2	0.6538	**0.9231**	0.9423
L1	0.6346	0.7885	0.9423
L2	**0.6731**	0.8654	**0.9808**
